# Surgical Resection vs. Percutaneous Ablation for Single Hepatocellular Carcinoma: Exploring the Impact of Li-RADS Classification on Oncological Outcomes

**DOI:** 10.3390/cancers13071671

**Published:** 2021-04-01

**Authors:** Leonardo Centonze, Stefano Di Sandro, Andrea Lauterio, Riccardo De Carlis, Samuele Frassoni, Antonio Rampoldi, Bruno Tuscano, Vincenzo Bagnardi, Angelo Vanzulli, Luciano De Carlis

**Affiliations:** 1 Department of General Surgery and Transplantation, Niguarda Ca’ Granda Hospital, 20162 Milan, Italy; stefano.disandro@unimore.it (S.D.S.); andrea.lauterio@ospedaleniguarda.it (A.L.); riccardo.decarlis@ospedaleniguarda.it (R.D.C.); luciano.decarlis@ospedaleniguarda.it (L.D.C.); 2 Hepato-Pancreato-Biliary Surgery and Liver Transplantation Unit, University of Modena and Reggio Emilia, 41124 Modena, Italy; 3 Department of Statistics and Quantitative Methods, University of Milan-Bicocca, 20126 Milan, Italy; samuele.frassoni@unimib.it (S.F.); vincenzo.bagnardi@gmail.com (V.B.); 4 Department of Diagnostic and Interventional Radiology, Niguarda Ca’ Granda Hospital, 20162 Milan, Italy; antoniogaetano.rampoldi@ospedaleniguarda.it (A.R.); bruno.tuscano@ospedaleniguarda.it (B.T.); angelo.vanzulli@ospedaleniguarda.it (A.V.); 5 School of Medicine and Surgery, University of Milan-Bicocca, 20126 Milan, Italy

**Keywords:** hepatocellular carcinoma, Li-RADS classification, surgical resection, percutaneous ablation, recurrence, survival

## Abstract

**Simple Summary:**

Li-RADS classification has recently emerged as an accurate tool for hepatocellular carcinoma diagnosis in the setting of liver cirrhosis, but its prognostic value has never been investigated so far. Single HCC benefits from both surgical resection and percutaneous ablation, although several studies support the superiority of surgery in terms of oncological results. We retrospectively and blindly classified 140 treatment-naïve single HCC according to Li-RADS protocol, comparing the oncological outcomes of surgical resection and percutaneous ablation for each Li-RADS subclass. Our analysis highlighted better overall survival, recurrence free survival and lower incidence of local recurrence after surgical resection in Li-RADS-5 nodules, while Li-RADS-3/4 subclasses showed similar outcomes after the two treatments. These results confirm the superiority of surgical approach for single HCC and suggest a potential prognostic role of Li-RADS classification, supporting liver resection especially for Li-RADS-5 subclass.

**Abstract:**

*Background*: Single hepatocellular carcinoma (HCC) benefits from surgical resection (SR) or US-guided percutaneous ablation (PA), although the best approach is still debated. We evaluated the impact of Li-RADS classification on the oncological outcomes of SR vs. PA as single HCC first-line treatment. *Methods*: We retrospectively and blindly classified treatment-naïve single HCC that underwent SR or PA between 2010 and 2016 according to Li-RADS protocol. Overall survival (OS), recurrence free survival (RFS) and local recurrence after SR and PA were compared for each Li-RADS subclass before and after propensity-score matching (PS-M). *Results*: Considering the general population, SR showed better 5-year OS (68.3% vs. 52.2%; *p* = 0.049) and RFS (42.5% vs. 29.8%; *p* = 0.002), with lower incidence of local recurrence (8.2% vs. 44.4%; *p* < 0.001), despite a significantly higher frequency of clinically-relevant complications (12.8% vs. 1.9%; *p* = 0.002) and a higher Comprehensive Complication Index (12.1 vs. 2.2; *p* < 0.001). Focusing on different Li-RADS subclasses, we highlighted better 5-year OS (67.1% vs. 46.2%; *p* = 0.035), RFS (45.0% vs. 27.0% RFS; *p* < 0.001) and lower incidence of local recurrence (9.7% vs. 48.6%; *p* < 0.001) after SR for Li-RADS-5 HCCs, while these outcomes did not differ for Li-RADS-3/4 subclasses; such results were confirmed after PS-M. *Conclusions*: Our analysis suggests a potential prognostic role of Li-RADS classification, supporting SR over PA especially for Li-RADS-5 single HCC.

## 1. Introduction

Hepatocellular carcinoma (HCC) represents the third cause of cancer-related death worldwide accounting for 85–90% of primary liver tumors, and is associated to liver cirrhosis in more than 80% of patients [1,2,3].

Up to the most recent guidelines from the European Association for the Study of the Liver (EASL) and the American Association for the Study of Liver Disease (AASLD), HCC diagnosis in the setting of liver cirrhosis relies on contrast-enhanced imaging [4,5].

American Association of Radiologist developed a standardized diagnostic algorithm for imaging reporting in the setting of liver cirrhosis: Liver Reporting & Data System (Li-RADS) [5]. The Li-RADS protocol provides HCC-specific diagnostic accuracy for each Li-RADS subclass, ranging from 37% in Li-RADS-3 up to 95% in Li-RADS-5 nodules [6,7], and has been recently included in AASLD guidelines for HCC management [5].

HCC treatment algorithms are deeply influenced by the correlation of HCC and underlying liver cirrhosis, as treatment options may be limited by the reduced physiological reserve of a diseased liver, as well as tumor burden.

Single HCC could benefit from both surgical resection (SR) or US-guided percutaneous ablation (PA) with similar oncological outcomes [8,9,10], although several studies advocate resection as the best treatment option [11,12,13]; notably, pathological analysis from surgical specimens could reveal the presence of unfavorable histological characteristics such as microvascular invasion or satellitosis, that may change future clinical decision making (i.e., favoring liver transplantation) [14,15,16]. Given this scenario, the aim of our study was to retrospectively evaluate the oncological impact of Li-RADS classification on single HCC treated by SR vs. PA.

## 2. Materials and Methods

Study protocol followed the 1975 Declaration of Helsinki ethical guidelines, as revised in Brazil in 2013. Local ethical committees’ review of the protocol deemed that formal approval was not required owing to the retrospective, observational and anonymous nature of this study. Results are reported according to Strengthening the Reporting of Observational Studies in Epidemiology (STROBE) [17].

### 2.1. Study Design

The study enrolled all adult patients (age ≥ 18 years) with a history of cirrhosis and a treatment-naïve single HCC without macrovascular invasion who underwent SR or PA as first-line treatment between 2010 and 2016, with an available pre-treatment contrast-enhanced imaging and >1-year follow-up.

All data were retrieved from a single university-affiliated, hepato-pancreato-biliary teaching center prospective database and anonymized prior to analysis.

A senior (A.V.) and a fellow (B.T.) radiologist retrospectively and blindly analyzed pretreatment contrast-enhanced imaging (either computed tomography or magnetic resonance imaging) in order to classify all nodules according to 2018 Li-RADS protocol, applying ancillary features when feasible [6]. HCCs whose edge was located 5 mm or less from the surface of the liver were defined as superficial.

Oncological outcomes of SR and PA were compared before and after Li-RADS stratification of the general population for Li-RADS-5 and Li-RADS-3/4 subclasses; such results were furtherly validated after propensity-score matching (PS-M).

### 2.2. Perioperative Management

HCC diagnosis followed EASL and AASLD guidelines [4,5]. All treatment options were discussed during multidisciplinary institutional boards composed by surgeons, hepatologists, diagnostic and interventional radiologists.

Impaired hepatic functional reserve (Child Pugh score C, ascites, platelet count < 50.000/uL) and high operative risk (ASA score > 3) were considered as contraindications to surgery; on the other hand, a peripheral exophytic lesion and a nodule diameter > 5cm or not detectable by ultrasound usually contraindicated ablation.

Postoperative or postprocedural complications were recorded according to Clavien-Dindo classification, and summarized in the Comprehensive Complication Index (CCI) [18,19], while post-operative biliary fistula and post-hepatectomy liver failure were defined following the International Study Group for Liver Surgery (ISGLS) classifications [20,21].

Liver transplantation was not considered as first line approach for early HCC, but represented the leading option in patients who experienced a transplantable recurrence [22]. All patients underwent life-long surveillance for HCC recurrence, and local recurrence was diagnosed and evaluated following the EASL guidelines [4].

### 2.3. Study Endopints

The endpoints of our study were recurrence-free survival (RFS), defined as the time from surgery or ablation until the date of any type of HCC recurrence, either local or distant, and overall survival (OS), defined as the time from surgery until the date of death, all causes considered.

### 2.4. Statistical Analysis

Continuous data are reported as median and interquartile ranges (IQR). Categorical data are reported as counts and percentages.

Comparisons between resected and ablated HCC patients were performed using the Wilcoxon test for continuous variables and Chi-square test for categorical variables.

Given the differences in the baseline characteristics between resected and ablated populations, and to control nonrandom assignment of the treatment, a second analysis was performed on a subset of all patients considered in the study, according to a PS-M.

The PS-M was estimated with the use of a multivariable logistic regression model, with treatment as the dependent variable and the following baseline characteristics as covariates: diameter of the nodule, alpha-fetoprotein (a-FP), satellitosis and platelet count. PS-M was performed with the use of a 1:1 matching without replacement (greedy-matching algorithm), with a caliper width equal to 0.30 of the propensity score. Comparisons between the two matched groups were performed using the Wilcoxon test for continuous variables and Fisher’s exact test for categorical variables.

The RFS and OS functions were estimated using the Kaplan-Meier method, and the log-rank test was performed to evaluate differences between groups.

The cumulative incidence functions (CIF) of local relapse were estimated according to method described by Kalbfleisch and Prentice, taking into account the competing causes of recurrence. Gray’s test was used to assess differences between groups.

A *p*-value < 0.05 was considered statistically significant for all analyses.

All analyses were performed with the statistical software SAS 9.4 (SAS Institute, Cary, NC, USA).

### 2.5. Surgical Technique

Liver resection was performed either open or laparoscopically according to patients and tumor characteristics; regardless of the chosen approach, all patients underwent intraoperative ultrasound for tumor and vascular mapping.

CUSA^®^ Excel/CUSA^®^ Excel + Cavitron Ultrasonic Surgical Aspirator System (Integra, Dublin, Ireland) and Ultracision Harmonic scalpel (Ethicon Endo-Surgery, Cincinnati, OH, USA) were used for parenchymal transection, while hemostasis and biliostasis on the liver cut surface were achieved using metallic clips, Hemolock or non-absorbable sutures.

Pedicle clamping was not routinely applied; when needed, it was performed intermittently, with no longer than 15 min of clamping time and at least 5 min of release [23].

### 2.6. Ablative Technique

The majority of ablated patients underwent percutaneous ultrasound-guided radiofrequency ablation, and selected cases (i.e., perivascular lesions) received microwave coagulation therapy: the most commonly used electrode was a LeVeen Needle Electrode with an insulated 17-gauge outer needle and retractable curved electrodes (model 70 and model 90 Starburst XL needles, RITA Medical Systems, Mountain View, CA, USA; LeVeen needle electrode, Boston Scientific, Boston, MA, USA), while some patients were treated by an internally cooled electrode (Cool-Tip RF Electrode; Radionics, Burlington, MA, USA).

## 3. Results

**Figure 1:**
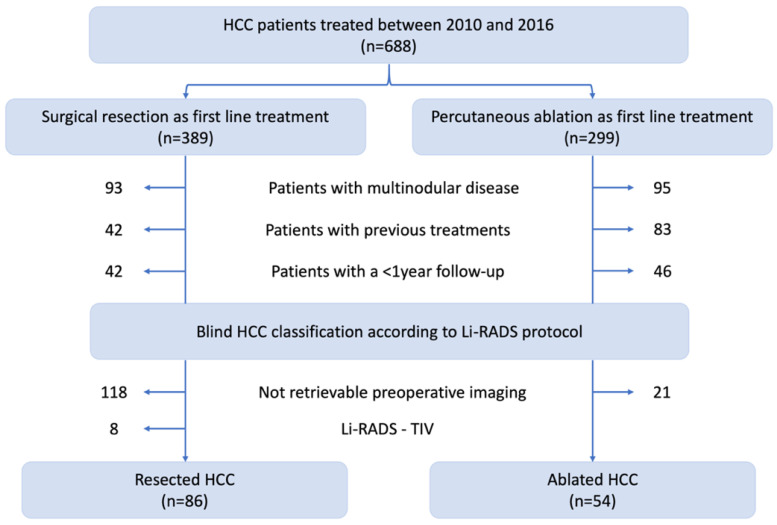
Flowchart for patient selection.

Our starting population was composed of 688 cirrhotic patients: of these, 389 underwent SR and 299 received PA as first-line treatment between 2010 and 2016. One-hundred-eighty-eight patients (93 resections and 95 ablations) were excluded as they presented multinodular disease. Other 125 patients (42 resections and 83 ablations) were excluded because of previous HCC diagnosis and/or treatment on past medical history. Eighty-eight patients (42 resections and 46 ablations) were excluded for a <1-year follow-up, so our population of 287 patients was composed of 75 ablated and 212 resected HCC. Preoperative imaging was not available for 118 resected and 21 ablated patients; 8 resected patients who presented macrovascular infiltration (Li-RADS-TIV) were furtherly excluded after imaging review. Our final population was composed of 140 patients: 86 resected and 54 ablated HCC. The workflow of patient selection is depicted in Figure 1.

### 3.1. Patients and HCC Characteristics

Clinical and demographical data of the study population are summarized in Table 1.

Resected patients presented lower bilirubin (0.71 mg/dL vs. 0.92 mg/dL; *p* = 0.013), lower INR (1.10 vs. 1.17; *p* = 0.001) and higher platelet count (159.000/μL vs. 98.000/μL; *p* < 0.001) compared to patients who underwent PA, while albumin-bilirubin (ALBI) and Child Pugh scores were not significantly different between the two treatment groups.

Patients who underwent SR had larger nodules (30 mm vs. 18 mm; *p* < 0.001) with higher prevalence of satellitosis, although not statistically significant (17.4% vs. 5.7%; *p* = 0.078); both a-FP levels and distribution of Li-RADS classes did not significantly differ between and resected and ablated patients. Table S1 depicts the type of liver resections that were performed [23].

### 3.2. Short-term Outcomes of PA and SR

Patients who underwent SR presented a higher incidence of clinically-relevant (Clavien-Dindo ≥ 3) complications (12.8% vs. 1.9%; *p* = 0.002) and higher CCI (12.1 vs. 2.2; *p* < 0.001) compared to patients treated by PA.

Six patients who underwent PA developed a grade 1–2 postprocedural complication according to Clavien-Dindo classification: three patients had fever and were treated with antibiotics; two patients developed gastroparesis and vomiting requiring prokinetics medications and one patient experienced prolonged pain related to diaphragmatic irritation and was treated with opioid analgesics.

One patient who underwent PA developed an hemoperitoneum requiring endovascular embolization, resulting in a Clavien-Dindo 3a complication.

Twenty-four patients in the SR group developed a Clavien-Dindo grade 1–2 complication: postoperative ascites occurred in eight patients, that required diuretics; five patients developed fever treated with antibiotics; four patients had a pleural effusion requiring diuretics and respiratory physiotherapy; three patients complained of nausea and vomiting, requiring prokinetics medications; two patients developed a mild pneumonia and two other patients had a superficial surgical site infection.

Nine patients who underwent SR developed a Clavien-Dindo grade 3–4 complication: two patients developed a deep surgical site infection treated by percutaneous drainage, resulting in a Clavien-Dindo 3a complication; an ISGLS grade B postoperative biliary fistula requiring endoscopic management occurred in two patients, resulting in a Clavien-Dindo 3a complication; three patients underwent relaparotomy for hemoperitoneum yielding a Clavien-Dindo 4a complication and two patients developed a grade C post-hepatectomy liver failure according ISGLS definition, resulting in a Clavien-Dindo 4b complication. Two fatalities occurred after SR, yielding in a 2.3% postoperative mortality.

Finally, SR resulted in a significantly longer hospital stay compared to PA (8 days vs. 4 days; *p* < 0.001).

### 3.3. Survival Analysis

Median follow-up lasted 5 years (IQR: 2.1–7.1 years) in the resected and 4.5 years (IQR: 3.1–5.4 years) in the ablated population. 

**Figure 2:**
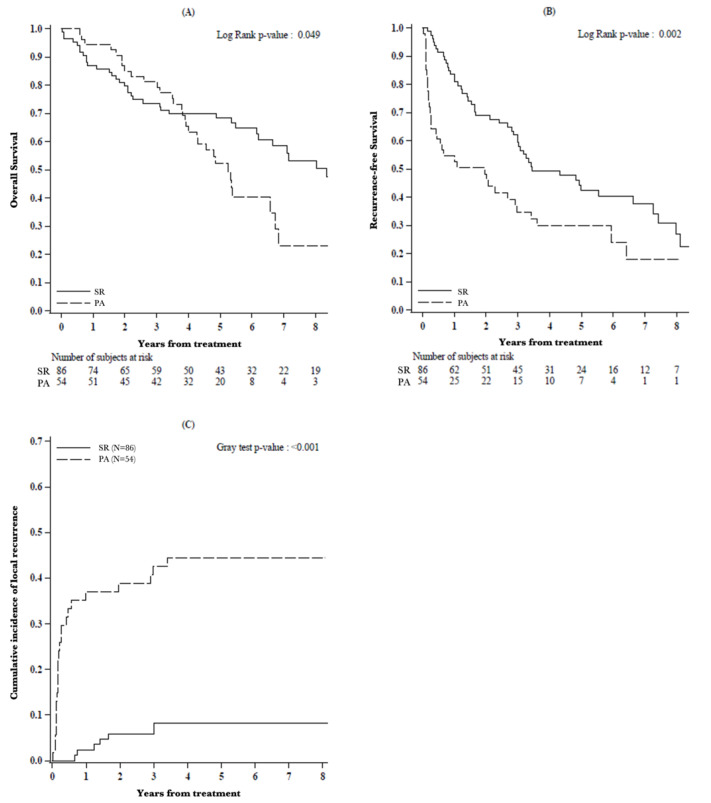
(**A**) Overall survival, (**B**) recurrence-free survival and (**C**) cumulative incidence of local recurrence by type of treatment (N = 140).

Considering the general population, SR offered significantly better OS (87.1%, 73.6% and 68.3% vs. 94.4%, 81.2% and 52.2% 1-, 3- and 5-year OS; *p* = 0.049), RFS (82.2%, 60.7% and 42.5% vs. 52.5%, 34.6% and 29.8% 1-, 3- and 5-year RFS; *p* = 0.002) and lower incidence of local recurrence (2.4%, 7.1% and 8.2% vs. 37.0%, 42.6% and 44.4% 1-, 3- and 5-year CIF of local recurrence; *p* < 0.001) compared to PA (Figure 2).

The management of local and intrahepatic recurrences that were diagnosed during the follow-up period are resumed in Figure S1 (after PA as first-line treatment) and Figure S2 (after SR as firs-line treatment).

### 3.4. Survival Analysis of Li-RADS Subclasses

**Figure 3:**
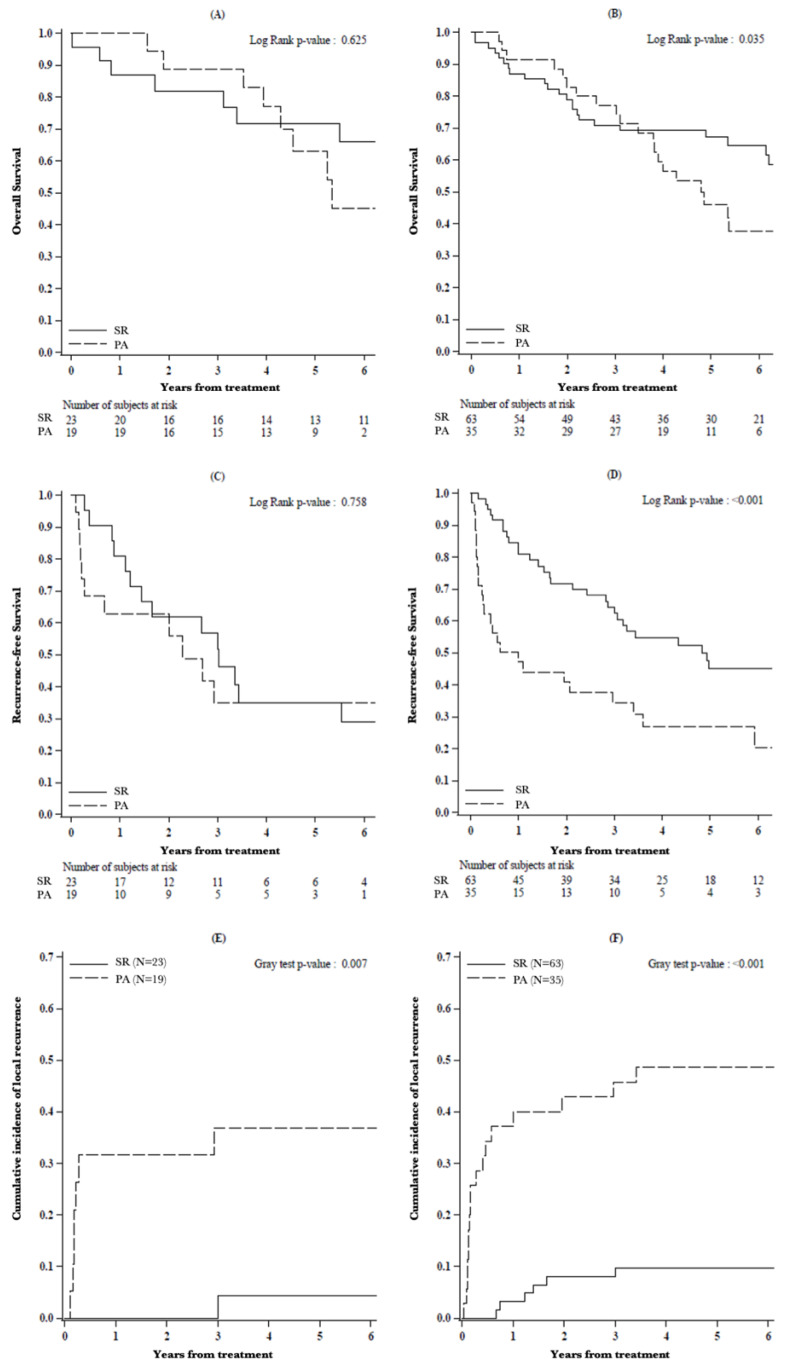
(**A**,**B**) Overall survival, (**C**,**D**) recurrence-free survival and (**E**,**F**) cumulative incidence of local recurrence in patients with nodule classified as (**A**,**C**,**E**) Li-RADS-3/4 and (**B**,**D**,**F**) Li-RADS-5, by type of treatment.

The second step of the analysis focused on oncological outcomes of SR and PA between two subsets of patients: those with a Li-RADS-3/4 and those with a Li-RADS-5 HCC (Figure 3).

Median follow-up lasted 5.9 years (IQR: 1.6–9.5 years) in the resected and 4.5 years (IQR: 3.5–5.4 years) in the ablated population.

OS did not significantly differ between resected vs. ablated patients in Li-RADS-3/4 subclasses (87.0%, 81.8% and 71.6% vs. 100.0%, 88.9% and 63.0% 1-, 3- and 5-year OS; *p* = 0.625) while it was significantly better in Li-RADS-5 HCC after SR (87.1%, 70.9% and 67.1% vs. 91.4%, 77.1% and 46.2% 1-, 3- and 5-year OS; *p* = 0.035).

The abovementioned statistically significant difference in RFS between resected and ablated patients was no longer evident in Li-RADS-3/4 (81.0%, 56.7% and 34.8% vs. 62.7%, 34.8% and 34.8% 1-, 3- and 5-year RFS; *p* = 0.758) HCCs, while it was retained in Li-RADS-5 subclass (82.7%, 62.3% and 45.0% vs. 47.1%, 34.2% and 27.0%1-, 3- and 5-year RFS; *p* < 0.001).

The incidence of local recurrence was significantly lower after SR for both Li-RADS-3/4 (0.0%, 0.0% and 4.3% vs. 31.6%, 36.8.6% and 36.8% 1-, 3-, and 5-year CIF of local recurrence; *p* = 0.007) and Li-RADS-5 nodules (3.2%, 9.7% and 9.7% vs. 40.0%, 45.7% and 48.6% 1-, 3- and 5-year CIF of local recurrence; *p* < 0.001).

### 3.5. Oncological Outcomes of PS-M Population

Median follow-up lasted 7.1 years (IQR: 3.4–9.6 years) in the resected and 5.3 years (IQR: 3.4–9.6 years) in the ablated population.

After PS matching, both OS (96.0%, 87.5% and 78.8% vs. 96.0%, 84.0% and 53.7% 1-, 3- and 5-year OS; *p* = 0.025), RFS (87.7%, 50.1% and 41.3% vs. 46.2%, 32.3% and 32.3% 1-, 3- and 5-year RFS; *p* = 0.049) and the incidence of local recurrence (0.0%, 12.0% and 12.0% vs. 48.0%, 56.0% and 56.0% 1-, 3- and 5-year CIF of local recurrence; *p* < 0.001) were significantly better after SR compared to PA (Figure S3).

Focusing on oncological results in different Li-RADS classes, we highlighted better OS (93.8%, 80.8% and 80.8% vs. 94.7%, 84.2% and 43.7% 1-, 3- and 5-year OS; *p* = 0.009), RFS (93.8%, 46.9% and 40.2% vs. 40.2%, 28.7% and 28.7% 1-, 3- and 5- year RFS; *p* = 0.038) and lower incidence of local recurrence (0.0%, 18.8% and 18.8% vs. 52.6%, 63.2% and 63.2%1-, 3- and 5-year CIF of local recurrence; *p* = 0.005) after SR in Li-RADS-5 nodules.

On the other hand, OS, RFS and CIF of local recurrence in Li-RADS-3/4 HCCs did not significantly differ between the two treatment groups (Figure S4).

### 3.6. Pathological Analysis of Resected Specimens

We achieved a R0 resection in 95% of HCCs. Focusing on the association between pathological features and Li-RADS subclasses, we documented a slightly higher frequency of G3 tumors (31.7% vs. 17.4%; *p* = 0.14), microvascular invasion (47.6% vs. 26.1%; *p* = 0.07) and satellitosis (20.6% vs. 8.7%; *p* = 0.33) in Li-RADS-5 nodules.

## 4. Discussion

The best approach to single HCC is still debated: several studies reported similar oncological outcomes after PA and SR [8,9,10], although many pieces of evidence support the superiority of surgery [11,12,13].

PA offers a minimally invasive approach that may grant acceptable oncological results with an average 5-year OS of 30–76% and a 5-year RFS of 14–49% [10,12,13,24,25,26,27]; compared to SR, PA shows better complication rates [13,28], but its oncological efficacy might be impaired by tumor location [11,29,30] and size [31,32].

On the other hand, SR offers a 5-year OS of 62–86% and a 5-year RFS of 41–82% [13,33,34,35], but bears higher complication rates compared to PA; moreover, SR could be precluded by impaired liver function that may limit extended parenchymal resections [34,36].

In this study we focused on a highly selected subset of patients presenting with a single treatment-naïve HCC without macrovascular invasion, approached by SR or PA.

As already highlighted in other series comparing surgery and ablation for HCC [11,12,13,28,34,35,36], SR showed an increased incidence of clinically-relevant complications, depicted by a higher CCI. Despite that, it should be noticed that the mean CCI of 12.2 in this surgical population of cirrhotic patients did not differ remarkably from the recently proposed ideal benchmarks of postoperative outcomes after liver resections [37].

Focusing on oncological outcomes, the analysis of our general population highlighted better OS, RFS and lower incidence of local recurrence after SR, confirming the results from other series [11,12,13].

Several studies pointed out that tumor size and location might affect the efficacy of ablative techniques: in fact, it has been proven that PA performs better in HCC nodules ≤ 3 cm, as documented by the 70% complete necrosis after histological analysis of transplant specimens [38]. Likewise, PA for subcapsular [39,40] or perivascular [41,42] tumors seem to achieve worse oncological results. On this behalf, it but must be noticed how mean tumor size of the ablated nodules was 18 mm and 20 mm before and after PS-M, respectively, and the distribution of tumor location did not significantly differ among the two treatment groups.

The impact of Li-RADS classification on the oncological outcomes of the two treatments in our unmatched population highlighted better OS and RFS in Li-RADS-5 nodules that underwent SR, while these differences were not evident for Li-RADS-3/4 classes. Likewise, our analysis showed higher incidence of local recurrence after PA regardless of Li-RADS classification.

We further performed a PS-M analysis, looking for stronger validation of the abovementioned results: such model was build considering the role of tumor size [43], satellitosis [44], a-FP levels [45] and liver function [46] on the oncological aggressiveness of HCC, in order to balance any confounding differences between the two treatment groups. Despite preliminary analysis of the baseline characteristics in pre-propensity population documented statistically significant higher INR and bilirubin levels in the ablated patients, these differences were not clinically relevant as their values were between the normal range in both treatment groups. On the other hand, the lower platelet count in PA group was considered as a significant indicator for portal hypertension, and incorporated in PS-M, as well as tumor related features such as size, a-FP and satellitosis.

After PS-M, we confirmed better OS, RFS and lower incidence of local recurrence after SR, documenting a deeper influence of Li-RADS classification on the oncological outcomes of the two treatments: in fact, SR resulted in better OS and RFS with lower incidence of local recurrence only for Li-RADS-5 nodules, while these outcomes did not differ for Li-RADS-3/4 subclasses.

These results might be explained by a more aggressive behavior of Li-RADS-5 HCCs compared to Li-RADS-3/4 nodules: such different outcomes could possibly rely on the association of Li-RADS-5 class with worse histological features. Despite the diagnostic accuracy of Li-RADS classification has been widely validated [7,47], up to now there are few reports focusing on its correlation with tumor differentiation and histology [48]. Even though the analysis on our resection specimens did not highlight any statically significant correlation between Li-RADS-5 class and unfavorable histological features, we depicted a higher frequency of these high-risk characteristics in Li-RADS-5 nodules, especially for microvascular invasion. Such radiological/histological correlation has never been investigated so far, and deserves further efforts on larger populations.

The main limitation of this study is represented by its retrospective nature, which may imply selection and indication biases, although Li-RADS classification did not influence neither patient selection nor management as it was retrospectively and blindly applied after treatment.

Another limitation is the relatively small study population, partially related to the loss of several cases for not-retrievable preoperative imaging (as many patients were referred by general practitioners or other centers after diagnostic workup); despite that, we should keep in mind that this analysis focused on a highly selected subclass of patients from a single center, which represented per se less than a half of the starting population.

## 5. Conclusions

This is the first analysis focusing on the impact of Li-RADS classification on the oncological results of SR vs. PA for single HCC that has been reported so far.

Despite a relatively higher complication rate after surgery, our findings support the superiority of SR over PA confirming the conclusions from other groups, and suggest a new potential role of Li-RADS classification in clinical decision making, shifting from a diagnostic to a prognostic tool.

Following these preliminary observations, SR should be especially supported in those patients bearing a Li-RADS-5 HCC in order to achieve better oncological results, whenever feasible. On the other hand, PA seems to grant similar outcomes compared to SR in Li-RADS-3/4 nodules, and should be considered as a valuable option for those cases requiring challenging surgeries or in less compensated patients.

Although promising, these findings should be cautiously applied to clinical practice, and several parameters (a-FP levels, liver functional reserve, tumor location) beside from Li-RADS subclass must always be evaluated in order to tailor the therapeutic approach to the individual patient and clinical context.

The results of this exploratory analysis should be verified on larger multicentric populations and hopefully validated by randomized cohort studies.

## Figures and Tables

**Table 1:** Patients’ characteristics before (N = 140) and after (N = 50) propensity score, by type of treatment.

Variable	Level	Before Propensity Score			After Propensity Score		
		SR (N = 86)	PA (N = 54)	*p* ^1^	SR (N = 25)	PA (N = 25)	*p* ^2^
Age (year), median (IQR)		67 (62, 74)	71 (59, 76)	0.37	66 (62, 73)	71 (59, 76)	0.64
MELD score, median (IQR)		8 (7, 9)	9 (8, 10)	0.071	8 (7, 9)	10 (7, 11)	0.062
PLT, median (IQR)		159 (109, 229)	98 (78, 139)	<0.001	126 (100, 159)	106 (79, 171)	*
ALT, median (IQR)		36 (26, 73)	43 (26, 86)	0.51	30 (23, 56)	49 (24, 91)	0.31
INR, median (IQR)		1.10 (1.04, 1.20)	1.17 (1.10, 1.33)	0.002	1.10 (1.06, 1.16)	1.19 (1.06, 1.38)	0.074
Bilirubin, median (IQR)		0.71 (0.50, 1.03)	0.92 (0.70, 1.28)	0.013	1.03 (0.64, 1.19)	0.82 (0.70, 1.13)	0.98
Albumin, median (IQR)		3.85 (3.42, 4.08)	3.80 (3.43, 4.07)	0.82	4.00 (3.49, 4.32)	3.80 (3.52, 4.10)	0.91
Nodule Size (mm), median (IQR)		30 (24, 45)	18 (15, 22)	<0.001	23 (20, 27)	20 (17, 24)	*
Sex, N (%)	Men	71 (82.6)	37 (68.5)	0.086	17 (68.0)	21 (84.0)	0.32
	Women	15 (17.4)	17 (31.5)		8 (32.0)	4 (16.0)	
Child score, N (%)	A	66 (86.8)	38 (79.2)	0.38	21 (87.5)	18 (75.0)	0.46
	B	10 (13.2)	10 (20.8)		3 (12.5)	6 (25.0)	
ALBI score, N (%)	Grade I	35 (40.7)	16 (32.7)	0.40	11 (44.0)	10 (40.0)	1.00
	Grade II	49 (57.0)	30 (61.2)		14 (56.0)	14 (56.0)	
	Grade III	2 (2.3)	3 (6.1)		0 (0.0)	1 (4.0)	
a-FP, N (%)	≤5	24 (34.3)	19 (39.6)	0.81	9 (36.0)	10 (40.0)	*
	>5–22	27 (38.6)	16 (33.3)		9 (36.0)	8 (32.0)	
	>22	19 (27.1)	13 (27.1)		7 (28.0)	7 (28.0)	
Presence of satellitosis, N (%)	No	71 (82.6)	51 (94.4)	0.074	23 (92.0)	23 (92.0)	*
	Yes	15 (17.4)	3 (5.6)		2 (8.0)	2 (8.0)	
Type of nodule, N (%)	Superficial	45 (52.3)	30 (55.6)	0.71	18 (72.0)	17 (68.0)	0.76
	Deep	41 (47.7)	24 (44.4)		7 (28.0)	8 (32.0)	
Li-RADS, N (%)	Li-RADS-3	8 (9.3)	7 (13.0)	0.56	5 (20.0)	3 (12.0)	0.68
	Li-RADS-4	15 (17.4)	12 (22.2)		4 (16.0)	3 (12.0)	
	Li-RADS-5	63 (73.3)	35 (64.8)		16 (64.0)	19 (76.0)	
**Outcomes**							
Hospital-stay (days), median (IQR)		8 (6, 12)	4 (3, 5)	<0.001	7 (6, 10)	3 (3, 5)	<0.001
Complications, N (%)	None	51 (59.3)	47 (87.0)	0.002	19 (76.0)	20 (80.0)	1.00
	Clavien 1–2	24 (27.9)	6 (11.1)		5 (20.0)	5 (20.0)	
	Clavien 3–5	11 (12.8)	1 (1.9)		1 (4.0)	0 (0.0)	
Comprehensive Complication Index	Mean (SD)	12.1 (22.5)	2.2 (6.4)	<0.001	3.8 (7.7)	3.4 (7.6)	0.81
	Median (IQR)	0 (0, 20.9)	0 (0, 0)		0 (0, 0)	0 (0, 0)	
R0	No	4 (4.7)	-	-	0 (0.0)	-	-
	Yes	82 (95.3)	-		25 (100.0)	-	

SR: surgical resection; PA: percutaneous ablation; IQR: interquartile range; PS: performance status.^1^ Wilcoxon p-value for continuous variables; Chi-square *p*-value for categorical variables; ^2^ Wilcoxon *p*-value for continuous variables; Fisher’s exact *p*-value for categorical variables; * Propensity score variables.

## Data Availability

Data will be available upon request to the corresponding author.

## Supplementary Materials

The following are available online at https://www.mdpi.com/article/10.3390/cancers13071671/s1, Table S1: Surgical technique before propensity score matching (N = 86); Figure S1: Management of local and intrahepatic recurrences after percutaneous ablation; Figure S2: Management of local and intrahepatic recurrences after surgical resection; Figure S3: (A) Overall survival, (B) recurrence-free survival and (C) cumulative incidence of local recurrence by type of treatment, among patients after propensity score (N = 50); Figure S4: (A,B) Overall survival, (C,D) recurrence- free survival and (E,F) cumulative incidence of local recurrence in patients with nodule classified as (A,C,E) Li-RADS-3/4 and (B,D,F) Li-RADS-5, by type of treatment, among patients after propensity score.
