# Supplementary material for: Surgical Resection vs. Percutaneous Ablation for Single Hepatocellular Carcinoma: Exploring the Impact of Li-RADS Classification on Oncological Outcomes
Source: Cancers (Basel). 2021 Apr 1;13(7):1671. doi: 10.3390/cancers13071671 (PMC8038048; doi:10.3390/cancers13071671)
Supplement: Supplementary file 1 [file cancers-13-01671-s001.pdf]

cancers

MDPI

Supplementary Materials

Surgical Resection vs. Percutaneous Ablation for Single Hepatocellular Carcinoma: Exploring the Impact of Li-RADS Classification on Oncological Outcomes

Leonardo Centonze [1,*], Stefano Di Sandro [1,2], Andrea Lauterio [1], Riccardo De Carlis [1], Samuele Frassoni [3], Antonio Rampoldi [4], Bruno Tuscano [4], Vincenzo Bagnardi [3], Angelo Vanzulli [4] and Luciano De Carlis [1,5]

Table S1. Surgical technique before propensity score matching (N=86).

Surgical Technique	N (%)
**Wedge (non-anatomic) resection**	53 (61.6)
**Segmentectomy**	14 (16.3)
**Bisegmentectomy**	6 (7.0)
**Left lobectomy**	8 (9.3)
**Right lobectomy**	5 (5.8)
**Laparoscopy**	29 (33.7)

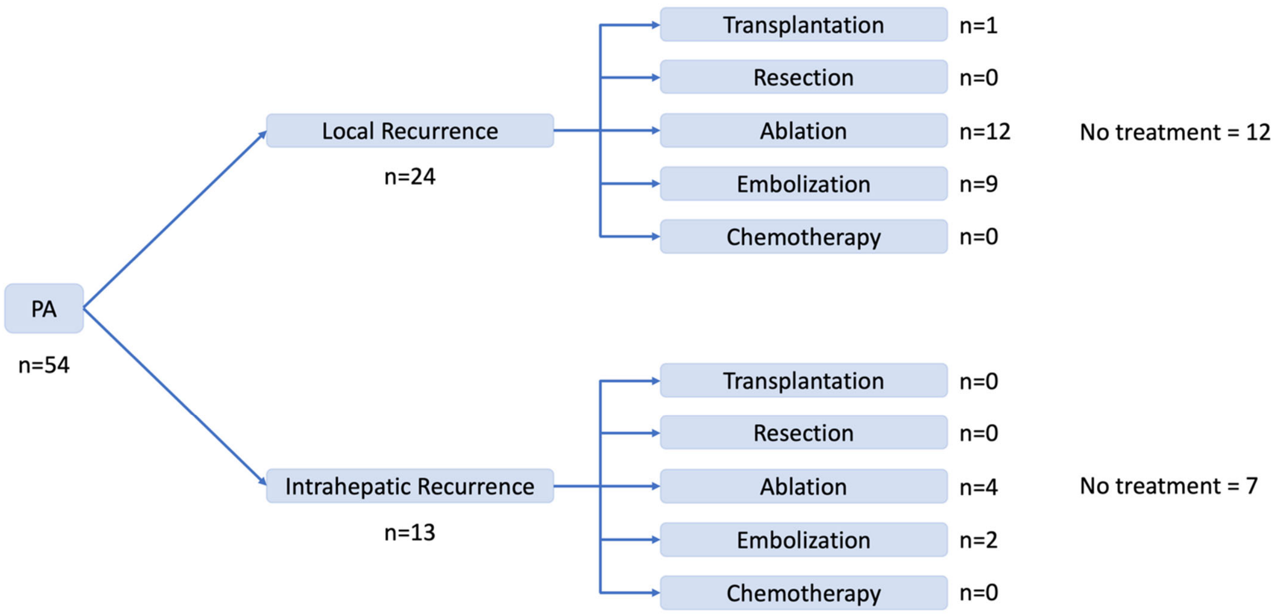

Figure S1. Management of recurrences after US-guided percutaneous ablation (PA) as first-line treatment.

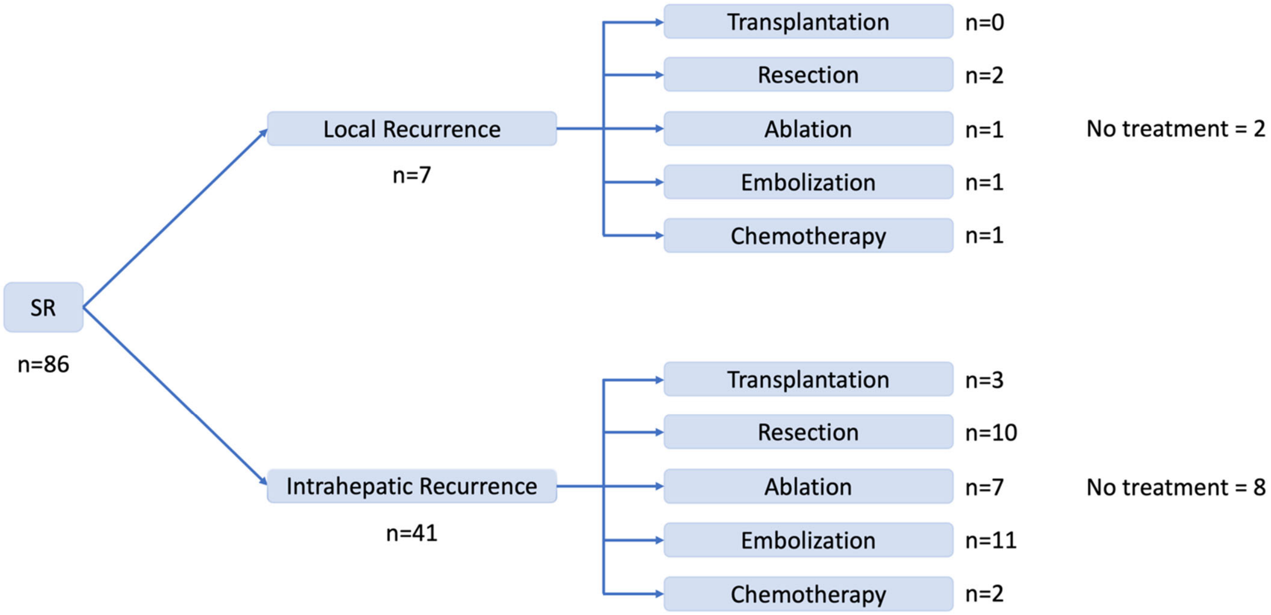

Figure S2. Management of recurrences after surgical resection (SR) as first-line treatment.

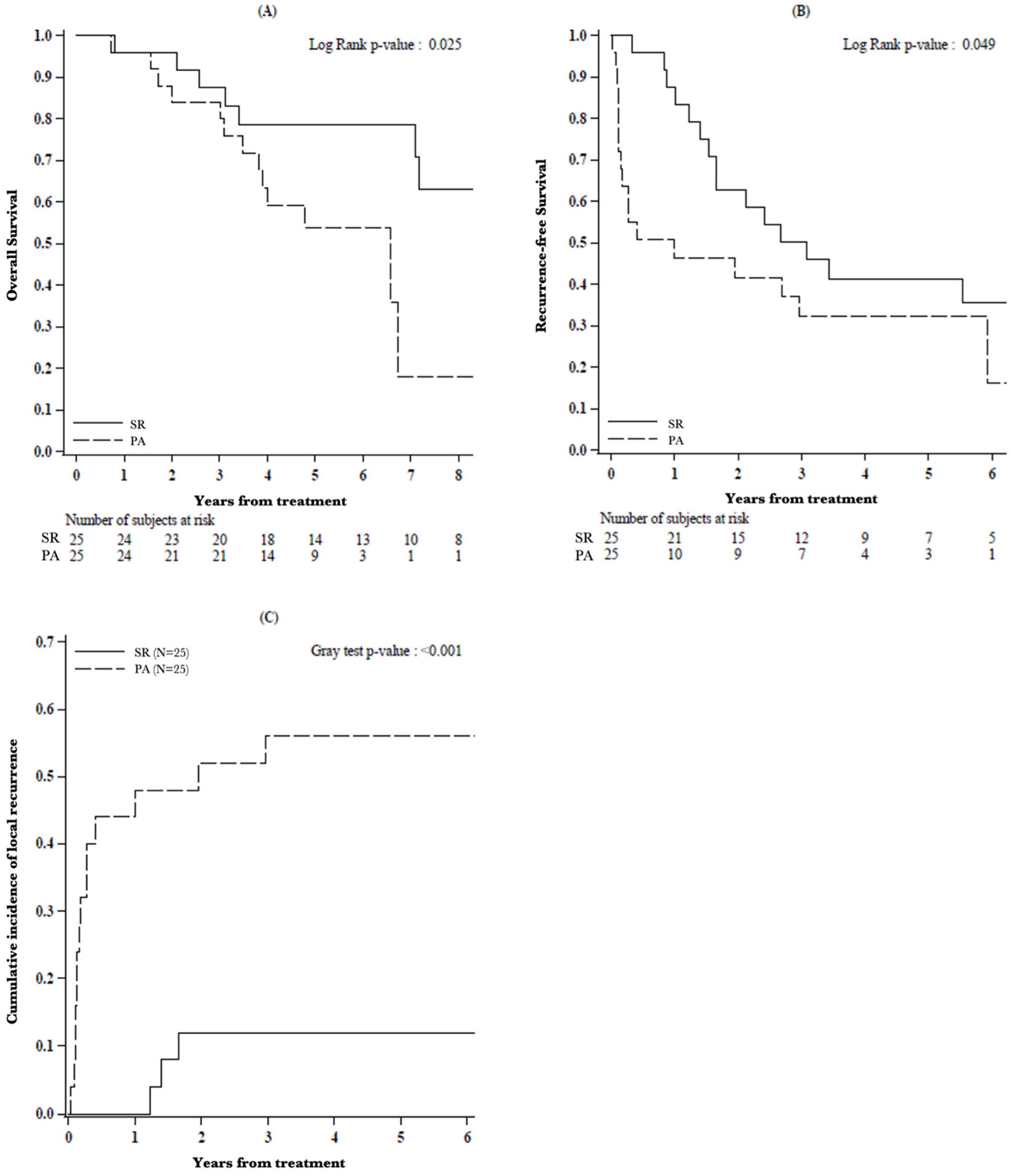

Figure S3. (A) Overall survival, (B) recurrence-free survival and (C) cumulative incidence of local recurrence by type of treatment, among patients after propensity score (N=50).

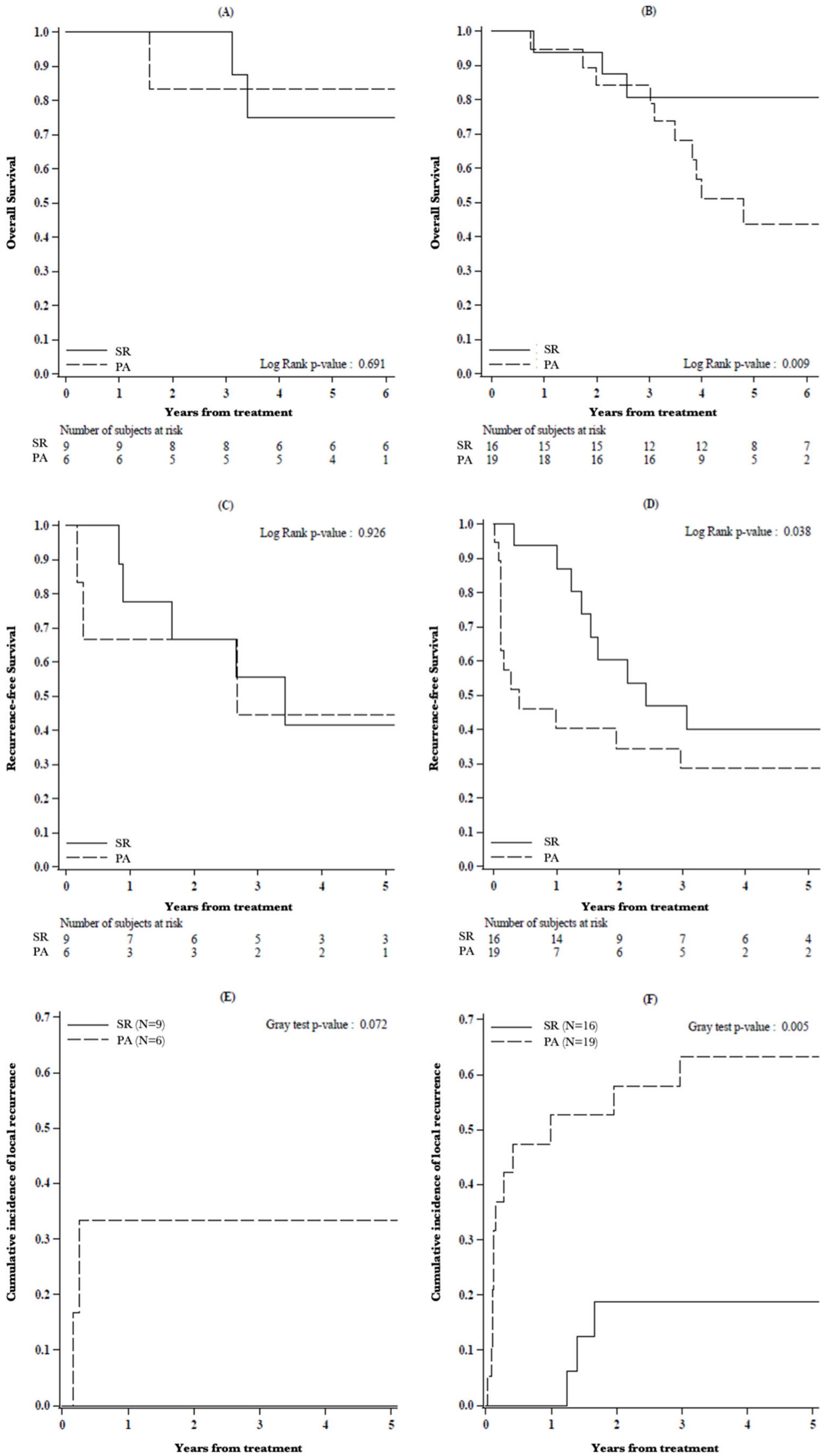

Figure S4. (A and B) Overall survival, (C and D) recurrence- free survival and (E and F) cumulative incidence of local recurrence in patients with nodule classified as (A, C and E) Li-RADS-3/4 and (B, D and F) Li-RADS-5, by type of treatment, among patients after propensity score.
